# Galectins-1, -3, and -7 Are Prognostic Markers for Survival of Ovarian Cancer Patients †

**DOI:** 10.3390/ijms18061230

**Published:** 2017-06-08

**Authors:** Heiko Schulz, Elisa Schmoeckel, Christina Kuhn, Simone Hofmann, Doris Mayr, Sven Mahner, Udo Jeschke

**Affiliations:** 1 Department of Gynaecology and Obstetrics, Ludwig-Maximilians University of Munich, Campus Großhadern: Marchioninistr. 15, 81377 Munich and Campus Innenstadt: Maistr. 11, Munich 80337, Germany; Heiko.Schulz@med.uni-muenchen.de (H.S.); Christina.Kuhn@med.uni-muenchen.de (C.K.); Simone.Hofmann@med.uni-muenchen.de (S.H.); Sven.Mahner@med.uni-muenchen.de (S.M.); 2 LMU Munich, Department of Pathology, Ludwig Maximilians University of Munich, Thalkirchner Str. 142, Munich 80337, Germany; Elisa.Schmoeckel@med.uni-muenchen.de (E.S.); Doris.Mayr@med.uni-muenchen.de (D.M.)

**Keywords:** Galectin-1, Galectin-3, Galectin-7, ovarian cancer, overall survival

## Abstract

There is a tremendous need for developing new useful prognostic factors in ovarian cancer. Galectins are a family of carbohydrate binding proteins which have been suggested to serve as prognostic factors for various cancer types. In this study, the presence of Galectin-1, -3, and -7 was investigated in 156 ovarian cancer specimens by immunochemical staining. Staining was evaluated in the cytoplasm and nucleus of cancer cells as well as the peritumoral stroma using a semi quantitative score (Remmele (IR) score). Patients’ overall survival was compared between different groups of Galectin expression. Galectin (Gal)-1 and -3 staining was observed in the peritumoral stroma as well as the nucleus and cytoplasm of tumor cells, while Gal-7 was only present in the cytoplasm of tumor cells. Patients with Gal-1 expression in the cytoplasm or high Gal-1 expression in the peritumoral stroma showed reduced overall survival. Nuclear Gal-3 staining correlated with a better outcome. We observed a significantly reduced overall survival for cases with high Gal-7 expression and a better survival for Gal-7 negative cases, when compared to cases with low expression of Gal-7. We were able to show that both tumor and stroma staining of Gal-1 could serve as negative prognostic factors for ovarian cancer. We were able to confirm cytoplasmic Gal-7 as a negative prognostic factor. Gal-3 staining in the nucleus could be a new positive prognosticator for ovarian cancer.

## 1. Introduction

Ovarian cancer is the most lethal gynecological malignancy, ranking fifth in estimated cancer deaths among women in the USA [1]. First-line treatment consists of primary debulking surgery followed by platinum and paclitaxel chemotherapy [2]. Still, the 5-year relative survival rate for epithelial ovarian cancer patients is less than 50% [3]. A lack of screening methods and the frequent presentation with advanced stage disease are considered as the main reasons for the poor outcome of ovarian cancer patients. Disease stage at diagnosis, extent of residual disease after surgery, histological subtype, and a high volume of ascites can be used as prognosticators in ovarian cancer [4]. Numerous studies have aimed to introduce new biological prognostic factors in ovarian cancer. Recently, carbohydrate stem cell marker TF1 has been proposed as negative prognostic marker in ovarian cancer displaying wildtype p53, while estrogen receptor promoter methylation could predict overall survival in low-grade ovarian carcinoma patients [5,6]. Although for these and various other molecules the prognostic value independently of clinical parameters has been proven, until today, except for breast cancer gene (BRCA)-status, no biological marker is commonly accepted [4]. Further specification of anti-cancer therapy necessarily requires an improvement of biological prognostic markers in ovarian cancer.

Galectins have been defined as a family of proteins sharing two main characteristics: a binding affinity for β-galactosides and a significant similarity in the carbohydrate-recognition domain (CRD) [7]. The first member of this family described was Galectin-1, which is isolated as homodimers composed of two identical CRD subunits [8]. Since then, the Galectin family has had a growing number of members, but only Galectin (Gal)-1–4, Gal-7–10, Gal-12, and Gal-13 are known to be present in humans [9]. Similar to Gal-1, Gal-7 typically occurs in homodimers, while Gal-3 is the only Galectin characterized chimeric protein known to form higher order oligomers [10,11]. In several cancer types, Galectins are known to affect tumor growth, metastasis, angiogenesis, cell migration, as well as tumor invasiveness and progression, and are therefore very likely to show a prognostic value for patients’ survival [9,12].

The role of Galectin-1 in cancer has been studied by various groups, and several papers already exist on this topic. For patients’ sera and ovarian cancer tissue, it has been shown that a combination of CA-125 and Galectin-1 serves as a possible two-marker combination for preoperative discrimination of benign and malignant ovarian masses [13]. Also, patients suffering from metastatic epithelial ovarian cancer were observed to show higher serum Gal-1 levels than those with non- metastatic type. Elevated peritumoral stroma staining of Gal-1 was shown to occur in advanced stages of epithelial ovarian cancer and is also connected with poorer progression-free survival in univariate analysis [14]. However, these results have not yet been reproduced for overall survival or confirmed by multivariate analysis [15]. Due to this, the possibility of Gal-1 as an independent prognostic marker in ovarian cancer still needs to be further investigated.

High cytoplasmic Galectin-3 expression has been suggested as a negative prognostic factor, as it was shown to correlate with shorter progression-free survival in ovarian cancer [16]. However, in another study, Gal-3 expression did not correlate to reduced overall survival, but a cytoplasmic staining pattern was associated with poor outcome when compared to patterns including nuclear staining [17]. Although Gal-3 staining in nucleus and stroma has been observed, their influence on overall survival still maintains elusive. Galectin-7 has been proposed to serve as negative prognostic factor in ovarian cancer by two independent groups. In both studies, its influence on progression-free survival and overall survival has been confirmed by univariate and multivariate analysis [16,18]. Yet, there is further disagreement whether Gal-7 staining occurs predominantly in the nucleus or the cytoplasm. Also, it remains unknown if there is a correlation between expressions of different Galectins in ovarian cancer, and there is a desperate need for comprehensive studies of various Galectins on a representative ovarian cancer panel. Therefore, in this study, we investigated the prognostic influence of Gal-1, -3, and -7 in patients with epithelial ovarian cancer and analyzed correlations to each other and to clinical and pathological parameters. We hypothesize that Gal-1, -3, and -7 are prognostic for overall survival in ovarian cancer patients, dependent of the localization of the Galectin expression.

## 2. Results

### 2.1. Gal-1 Tumor and Stroma Staining Is Negative Prognostic for Overall Survival

**Figure 1:**
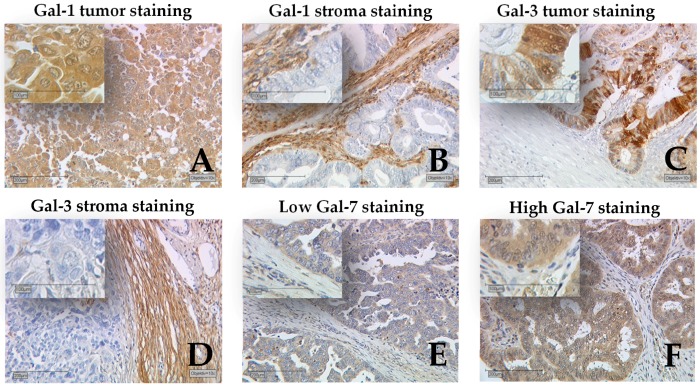
Detection of Galectins by immunohistochemistry. Representative photomicrographs are shown. Galectin (Gal)-1 was present in the cytoplasm and the nuclei of ovarian cancer cells (**A**) as well as the peritumoral stroma (**B**); Gal-3 staining was observed in the nuclei, cytoplasm (**C**), and stroma (**D**); Staining for Galectin-7 was mainly observed in the cytoplasm (**E**); only a few individual cases showed nuclear staining (**F**); 10× magnification was used for the outer pictures and 50× magnification for the inserts. The scale bars in in the outer pictures equal 200 μm (10× magnification) and the scale bars in the inserts equal 100 μm (50× magnification).

Galectin-1 staining was successfully performed on 150 ovarian cancer specimens. Gal-1 was present in the cytoplasm and the nuclei of ovarian cancer cells, as well as the peritumoral stroma (Figure 1). In 102 cases (68.0%), the cytoplasms of tumor cells were positive for Gal-1, with a median Remmele score (IRS) of 3. Peritumoral stroma was positive for Gal-1 in 148 cases (98.0%), with a median IRS of 8. Gal-1 expression significantly correlated with several clinical and pathological data (Table 1).

Gal-1 staining in cytoplasm and nucleus showed differences for several histological subtypes (*p* = 0.008, *p* = 0.002, respectively). Cytoplasmic Gal-1 staining was significantly stronger in serous, clear cell, or endometrioid subtypes, while for mucinous subtype we found more negative cases. Also, more cases showed Gal-1 positive nuclei for serous and clear cell subtypes, while endometrioid and mucinous subtypes had weaker nuclear Gal-1 stainings. Furthermore, Gal-1 staining in nucleus, cytoplasm, and stroma were significantly higher in cases with advanced tumor stage (*p* < 0.001, *p* = 0.006, *p* = 0.02, respectively). Gal-1 expression in the cytoplasm was significantly higher in cases with higher grading (*p* < 0.001) and advanced FIGO (Fédération Internationale de Gynécologie et d’Obstétrique) stage (*p* = 0.001). Gal-1 staining in the nucleus showed higher IR scores in lymph node positive cases (*p* = 0.001) and cases with advanced FIGO stage (*p* = 0.013).

**Figure 2:**
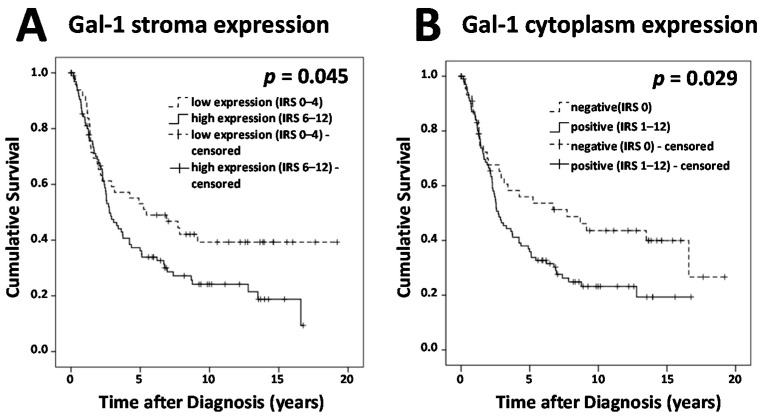

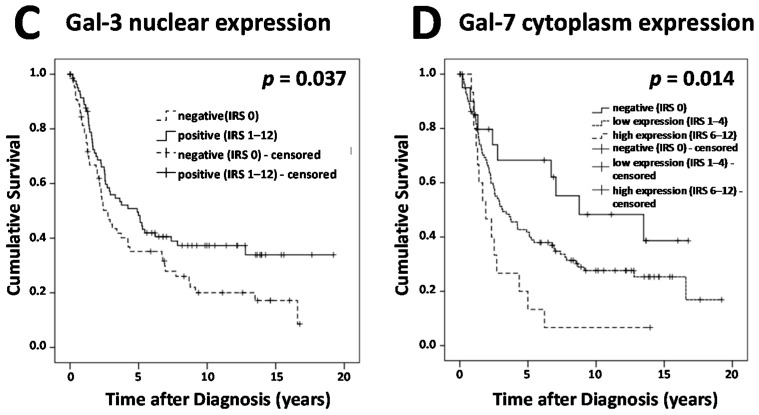
Survival times were plotted as Kaplan-Meier graphs. Percentage of living patients (vertical axis) was plotted in dependence of time (horizontal axis). Patients without an observed event (death) who exited the study before the observation period ended have been censored. Censoring has been marked in the graphs. Survival times of different groups of Galectin expression have been compared. Cases displaying high Gal-1 expression in the stroma showed a significantly reduced outcome compared to cases with low Gal-1 expression in the stroma. (**A**) Cases with Gal-1 expression in the cytoplasm showed significantly reduced overall survival compared to cases without any Gal-1 expression in cytoplasm; (**B**) Cases without Gal-3 expression in nuclei showed significantly reduced overall survival compared to cases with nuclear Gal-3 expression; (**C**) Cases with high Gal-7 expression showed a significantly reduced overall survival and Gal-7 negative cases showed better overall survival, when compared to cases with low expression of Gal-7; (**D**) Galectin expression was determined in cytoplasm, nucleus, and stroma using Remmele (IR) scores.

Survival times of different groups of Gal-1 expression in nucleus, cytoplasm, and stroma have been compared (Figure 2). Cases with Gal-1 expression in the cytoplasm showed significantly reduced overall survival compared to cases without any Gal-1 expression in the cytoplasm (*p* = 0.029) Moreover, cases displaying high Gal-1 expression in the stroma showed a significantly reduced outcome compared to cases with low Gal-1 expression in the stroma (*p* = 0.045). Comparing negative versus positive cases of Gal-1 expression in the nucleus did not show any differences with regard to overall survival. However, based on considering a multivariate analysis, only Gal-1 stroma staining would serve as an independent prognostic factor (Table 2).

### 2.2. Presence of Gal-3 in Nuclei Is A Positive Prognosticator in Ovarian Cancer

Gal-3 positive nuclei were observed in 83 (55%) out of 151 cases, while 96 cases (63.6%) showed cytoplasmic Gal-3 staining and 85 cases (56.3%) presented with Gal-3 positive peritumoral stroma (Figure 1). Median IR scores for Gal-3 in nuclei, cytoplasm, and stroma were 1, 2, and 1, respectively. Gal-3 staining showed correlations with clinical and pathological data (Table 3). Gal-3 expression in stroma and nucleus was different for several histological subtypes (*p* = 0.008, *p* = 0.013, respectively). Gal-3 stroma staining was stronger in serous and clear cell subtypes but weaker in endometrioid and mucinous subtypes, while nuclear Gal-3 staining was stronger in serous, clear cell, and mucinous subtypes but weaker in endometrioid subtype. Tumors rated as pT1 presented with significantly stronger nuclear Gal-3 staining than pT2 or higher staged cases (*p* = 0.042). We observed a correlation of Gal-3 in nucleus and cytoplasm with patients′ age (*p* = 0.022, *p* = 0.013, respectively), with higher IR scores for patients younger than 60 years. For our study panel, Gal-3 overexpression in the cytoplasm was not correlated with poorer outcome of ovarian cancer patients. Also, Gal-3 staining in the peritumoral stroma could not serve as a prognostic factor. However, nuclear Gal-3 expression could serve as a positive prognostic factor (Figure 2). Cases without Gal-3 expression in nuclei showed significantly reduced overall survival compared to cases with nuclear Gal-3 expression (*p* = 0.034). According to the results of a multivariate analysis, nuclear Gal-3 staining could not serve as an independent prognostic factor, probably due to its strong correlations with patients’ age, tumor stage, and histology (Table 2).

### 2.3. Gal-7 Expression Level Predicts Shortened Overall Survival in Ovarian Cancer

Staining for Galecin-7 was mainly observed in the cytoplasm; only few individual cases showed nuclear staining (Figure 1). Cytoplasmic Gal-7 staining was present in 129 (86.6%) out of 149 specimens, with a median IR score of 3. In total, 20 cases presented negative for Gal-7, while 114 cases showed low and 15 cases showed high expression of Gal-7. Gal-7 expression appeared to show differences for several histological subtypes (*p* = 0.026). The strongest Gal-7 staining was found in serous subtype, and the weakest was in endometrioid subtype (Table 4). No other correlation of Gal-7 with pathological data was found. Survival times of Gal-7 negative cases and cases displaying a high Gal-7 expression were compared to cases with low Gal-7 expression (Figure 2). We observed a significantly reduced overall survival for cases with high Gal-7 expression and a better survival for Gal-7 negative cases, when compared to cases with low expression of Gal-7 (*p* = 0.014). Also, according to the results of a multivariate analysis, higher Gal-7 expression can be confirmed as an independent prognostic factor for overall survival in ovarian cancer (Table 2).

### 2.4. Correlation Analysis

A correlation analysis is shown in Table 5. For Gal-1 staining, we observed positive correlations between staining in cytoplasm, nucleus, and stroma. Also, staining results of Gal-3 in cytoplasm, nucleus, and stroma were positively correlated among each other. Furthermore, we found correlations between Galectin-1 and -3 staining in nucleus, cytoplasm, and stroma. Gal-7 staining showed positive correlations with Gal-1 in cytoplasm and nucleus and all types of Gal-3 staining.

## 3. Discussion

According to our data, Gal-1 staining in cytoplasm and stroma share a negative prognostic impact on overall survival in ovarian cancer. In accordance, in vitro experiments showed that overexpression of Galectin-1 significantly increases migrative and invasive behavior of ovarian cancer cells [19]. Furthermore, Gal-1 knockdown experiments in ovarian cancer cells displayed a reduction in cell growth, migration, and invasion. Gal-1 interaction with H-Ras and activation of the Raf/extracellular signal-regulated kinase (ERK) pathway as well as the downregulation of matrix metalloproteinase-9 (MMP-9) and c-Jun could have been explored as possible mechanisms. Moreover, Gal-1 overexpression could significantly decrease the sensitivities of ovarian cancer cells to cisplatin, illustrating a possible explanation for decreased survival of ovarian cancer patients with increased Gal-1 expression [14]. Thus, Gal-1 is a promising new target for ovarian cancer therapy. For this purpose, several compounds targeting Gal-1 have been introduced [20]. OTX008, for instance, a new compound binding non-covalently to Gal-1 on the side back face, was able to inhibit proliferation and invasion of various cancer cells lines [21]. Anti-proliferative effects of OTX008 correlated with Gal-1 expression across a large panel of cell lines. Moreover, OTX008 efficiently inhibited the growth of ovarian cancer xenografts in vivo [22]. According to the results of a multivariate analysis, only Gal-1 stroma staining could serve as independent prognostic factor. Accumulation of Gal-1 in peritumoral stroma has been described for various other tumor entities [23,24,25]. Some groups tried to investigate the mechanisms responsible for this phenomenon. In situ hybridization experiments were able to show that fibroblast cells, adjacent to malignant cells, express Gal-1 mRNA, illustrating a possible explanation for peritumoral Gal-1 accumulation. Also, it was demonstrated that ovarian cancer cells produce Gal-1 and release it to the medium. Furthermore, conditioned medium obtained from ovarian carcinoma cells is able to induce increased gal-1 expression in fibroblast cells. Both experiments suggest that primarily the ovarian cancer cells might be responsible for stromal Gal-1 expression [26]. Our exploration of the positive correlation between Gal-1 staining in peritumoral stroma and malignant cells is consistent with this hypothesis. However, it requires further investigations to explain cases without Gal-1 expression in cancer cells but in the stroma or vice versa.

Several groups have suggested that higher Gal-3 expression is associated with reduced progression-free survival in ovarian cancer [17,27]. However, in these studies, observation of Gal-3 expression was limited to the cytoplasm, while the prognostic value of nuclear Gal-3 staining has not been further studied. We could not confirm a negative influence of cytoplasmic Gal-3 overexpression on overall survival for our study panel. On the contrary, nuclear Gal-3 staining served as a positive prognostic factor, although not independent of clinical and pathological parameters. Apparently, it is the nuclear and not cytoplasmic Gal-3 expression that has a major influence on patients’ outcome. In line with this, Gal-3 has been observed to play an important role in nuclear cell physiology, as it is involved in the mechanisms of pre-mRNA-splicing or mRNA transport [28,29]. Furthermore, cell culture experiments using human cervix adenocarcinoma HeLa-cells showed a delayed DNA damage repair response activation and a decrease in the G2/M cell cycle checkpoint arrest in the absence of Gal-3 [30]. A similar mechanism could be conceivable in ovarian cancer, predisposing cells for further mutations in the absence of nuclear Gal-3. To our knowledge, reduced Gal-3 expression as an indicator of poorer prognosis has only been observed in gastric cancer so far [31]. In cholangiocarcinoma, Gal-3 expression was associated with a poorly-differentiated type, while in vitro experiments showed significantly increased cell migration and invasion after suppression of Gal-3 expression [32]. However, for ovarian cancer, in vitro experiments showed knockdown of Gal-3 inhibits migration and invasion of cancer cells, while apoptosis and sensitivity to carboplatin increases [33]. Moreover, paclitaxel and additional Gal-3 inhibitor treatment showed synergistic cytotoxic effects and increased apoptosis in an on ovarian cancer cell line [34]. Since there are disagreements in previous research and our data is neither consistent with previous studies on progression-free survival nor with recent results of in vitro research, further investigation on the prognostic role of Gal-3 in ovarian cancer is definitely required.

As recently proposed by other groups, we were able to confirm Gal-7 as negative prognosticator for overall survival in ovarian cancer in uni- and multivariate analysis. Further cell culture experiments were able to prove that Gal-7 expression is induced by a mutant form of p53. Also, gal-7 was shown to increase proliferation [16], invasiveness, and motility of ovarian cancer cells, while interacting immunosuppressive by killing Jurkat T-cells and human peripheral T-cells [18]. All in all, these investigations confirm Gal-7 as a new promising target for specific therapeutic option in epithelial ovarian cancer. We observed various positive correlations between Gal-1, -3, and -7. This observation, and the fact that Galectins share binding affinities and have similarities in protein structure, suggests the assumption that Galectins might also share common functions in ovarian cancer molecular biology. However, since this observation is rather descriptive, further investigations are required to explore the biological characteristics and functions of different Galectins to determine the manner(s) in which they are similar or different in specific regards to their role(s) in ovarian cancer.

## 4. Materials and Methods

### 4.1. Patients

Formalin-fixed, paraffin-embedded (FFPE) ovarian cancer samples from 156 female patients who underwent surgery at the Department of Obstetrics and Gynecology, Ludwig-Maximilians- University of Munich, Germany between 1990 and 2002 were analyzed in this study. Women diagnosed for benign or for borderline tumors of the ovary were excluded and no patient had received neo-adjuvant chemotherapy. Tumor grading (G1 (*n* = 38), G2 (*n* = 53), G3 (*n* = 53)), and histological characterization (serous (*n* = 110), endometrioid (*n* = 21), clear cell (*n* = 12), mucinous (*n* = 13)) were performed by a gynecological pathologist. Tumor staging was accomplished using FIGO classification (I (*n* = 35), II (*n* = 10), III (*n* = 103), IV (*n* = 3)). TNM classification was performed according to UICC. Data on the extension of the primary tumor was available in 155 cases (T1 (*n* = 40), T2 (*n* = 18), T3 (*n* = 93), T4 (*n* = 4)), data on lymph node involvement was available in 95 cases (N0 (*n* = 43), N1 (*n* = 52) and data on the presence of distant metastasis was available in 9 cases (M0 (*n* = 3), M1 (*n* = 6). Clinical data was retrieved from patients’ charts and follow up data was requested from the Munich Cancer Registry. Patients’ age at surgery ranged between 31 and 88 years, with a median age of 62 ±12 years. Mean overall survival was 3.2 ± 3.0 years and 104 deaths were observed in total. The mean follow up time was 5.1 ± 4.8 years.

### 4.2. Immunochemistry

Resected ovarian cancer tissue samples were fixed in formalin and embedded in paraffin after surgery. For histopathological investigations, sections were dewaxed in Xylol for 20 minutes and immersed in 3% hydrogen peroxide (Merck, Darmstadt, Germany) to quench endogenous peroxidase. Then slides were rehydrated in a descending series of alcohol (100%, 75%, and 50%), and cooked for 5 minutes in sodium citrate buffer (0.1 mol/L citric acid/0.1 mol/L sodium citrate, pH 6.0) in a pressure cooker to ensure epitope retrieval. Afterwards, slides were washed in distilled water and phosphate-buffered saline (PBS), followed by a specific procedure for each Galectin staining. In particular, for Galectin-1 (Gal-1) staining, slides were blocked using power block (BioGenex, San Ramon, CA, USA) for 3 min at room temperature and incubated with Anti-Galectin 1 primary antibody (goat, polyclonal; R&D Systems, Minneapolis, MN, USA) at a final concentration of 0.033 µg/mL in power block (BioGenex, San Ramon, CA, USA) for 16 h at 4 °C. Galectin-3 (Gal-3) staining was performed by blocking specimens with 1.5% horse serum (Vector Laboratories, Burlingame, CA, USA) for 30 min at room temperature and incubating with Anti-Galectin 3 primary antibody (mouse, monoclonal, Novocastra Reagents, Leica Biosystems, Wetzlar, Germany) at a final concentration of 4.6 µg/mL in PBS for 16 h at 4 °C. For Galectin-7 (Gal-7) staining, specimens were blocked with Blocking Solution (Reagent 1; ZytoChem Plus HRP Polymer System (Mouse/Rabbit); Zytomed Systems GmbH, Berlin, Germany) for 5 minutes at room temperature. Slides were then incubated with Anti-Gal-7 (rabbit, polyclonal; Abcam, Cambridge, UK) at a final concentration of 2.5 µg/mL in PBS for 16 h at 4 °C. Afterwards, for Gal-1 and -3 staining, slides were incubated with isotype-matching anti-goat/mouse-IgG secondary antibody and avidin-biotin-peroxidase complex both for 30 min at room temperature, according to ABC Vectastain kit (Vector Laboratories, Burlingame, CA, USA). For Gal-7 staining, specimens were incubated in post-block reagent (Reagent 2) (Zytomed Systems GmbH, Berlin, Germany) and HRP-Polymer (Reagent 3) (Zytomed Systems GmbH, Berlin, Germany) for 30 min at room temperature, according to the manufacturer’s protocol (ZytoChem Plus HRP Polymer System (Mouse/Rabbit). All slides were washed twice in PBS for 2 min after every incubation step. For visualization reaction, every specimen was stained with 3,3′-diaminobenzidine chromogen (DAB; Dako, Glostrup, Denmark), stopped after 30 s to 2 min with tap water, counterstained in Mayer acidic hematoxylin, dehydrated in an ascending series of alcohol followed by xylol, and covered with Consul Mount (Thermo Shandon, Pittsburgh, PA, USA). Tissue sections that had been previously incubated with isotype-matched rabbit-/mouse-/goat- IgG (Dako, Hamburg, Germany) instead of the primary antibody served as negative controls. For positive control, tissue slides of placental tissue (Gal-1, -3) or breast cancer (Gal-7) were used. Primary antibodies were chosen due to high expected staining specificities according to the results of positive control staining, description, and example pictures on the manufacturer’s homepages. A semi-quantitative method (IR score; Remmele score) was performed by two independent observers in consensus to obtain staining results. For this purpose, the predominant staining intensity (0 = negative, 1 = low, 2 = moderate, and 3 = strong) and the percentage of stained cells (0 = 0%, 1 = 1–10%, 2 = 11–50%, 3 = 51–80%, and 4 = 81–100% stained cells) has to be multiplied, resulting in values from 0 to 12. Staining intensity was measured in the cytoplasm and the nucleus of the cancer cells, and in the peritumoral stroma. Cut-off points for IR scores were chosen specifically for each staining with regard to the distribution pattern of IR scores in the collective. For Gal-1 staining in cytoplasm and nucleus of cancer cells, IRS = 0 was considered as negative and an IRS ≥ 1 as positive. For stroma staining, Gal-1 groups of low expression (IRS < 5) and high expression (IRS ≥ 5) were compared. For analysis of Gal-3 staining, negative cases with an IRS = 0 were compared to positive cases with an IRS ≥ 1. Gal-7 expression was grouped as negative (IRS = 0), low (1 ≥ IRS ≥ 4), and high (IRS ≥ 6).

### 4.3. Statistical Analysis

Statistical data was obtained using SPSS 23.0 (v23, IBM, Armonk, NY, USA) statistic software. Distribution of clinicopathological variables was tested with Chi-Square Statistics. Mann-Whitney *U*-test was used to compare IR scores of Galectins between different clinical and pathological subgroups. Correlations between immunochemical staining results were calculated using Spearman’s correlation analysis. Kaplan-Meier curves and Log-rank test (Mantel-Cox) were used to compare survival times between different groups. Data are presented with the mean ± standard deviation. Values of *p* < 0.05 were considered as significant.

### 4.4. Ethics Statement

All tissue samples used for this study were left-over material from the archives of LMU Munich, Department Gynecology and Obstetrics, Ludwig-Maximilians-University, Munich, Germany, that had initially been collected for histopathological diagnostics. All diagnostic procedures had already been fully completed at the time the histopathological investigations for the current study were performed. Patients’ data have been fully anonymized. The study was approved by the Ethics Committee of LMU Munich. All experiments were performed according to the standards set in the declaration of Helsinki 1975.

## 5. Conclusions

We were able to show that Galectin expression and its impact on overall survival of ovarian cancer patients is strongly dependent of its localization, whether it is in the nucleus or cytoplasm of tumor cells or the peritumoral stroma. We elaborated that Gal-1 tumor and stroma staining, and Gal-7 staining in the cytoplasm serves as a negative prognostic factor for overall survival in ovarian cancer, while nuclear Gal-3 staining could serve as a positive prognostic factor. According to the results of a multivariate analysis, Gal-1 stroma staining and Gal-7 staining are prognostic factors, independent of clinical and pathological parameters.

## Figures and Tables

**Table 1:** Gal-1 staining correlated with clinical and pathological data.

Clinical and Pathological Variables	Gal-1 Expression Cytoplasm		*p*	Gal-1 Expression Stroma		*p*	Gal-1 Expression Nucleus		*p*
	negative	positive		low	high		negative	positive	
Histology									
Serous	26	79	0.008	34	71	NS	27	78	0.002
Clear cell	5	7		6	6		3	9	
Endometrioid	8	12		7	13		11	9	
Mucinous	9	4		3	10		9	4	
Tumor Stage									
pT1	22	17	<0.001	20	19	0.006	19	20	0.020
pT2+	26	84		30	80		31	79	
Lymph node									
pN0/pNX	36	65	NS	34	67	NS	43	58	0.001
pN1	12	37		16	33		7	42	
Distant Metastasis									
pM0/pMX	47	97	NS	49	95	NS	49	95	NS
pM1	1	5		1	5		1	5	
Grading									
G1	20	16	<0.001	13	23	NS	14	22	NS
G2+	22	80		31	71		31	71	
FIGO									
I/II	22	21	0.001	17	26	NS	21	22	0.013
III/IV	24	78		31	71		28	74	
Age									
≤60 years	27	52	NS	28	51	NS	24	55	NS
≤60 years	21	50		22	49		26	45	

TNM staging was accomplished according to actual standards of Union for International Cancer Control (UICC); pT1 = tumor stage 1; pT2+ = tumor stage 2 or higher; pN0 = lymph node stage 0; pNX = lymph node stage not evaluated; pN1 = lymph node stage 1; pM0 = distant metastasis stage 0; pMX = distant metastasis not evaluated; pM1 = distant metastasis stage 1; G1 = grade 1; G2+ = grade 2 or higher; FIGO = Fédération Internationale de Gynécologie et d’Obstétrique; NS = Not significant (*p* > 0.05).

**Table 2:** Multivariate analysis.

Covariate	Coefficient (b_i_)	HR Exp (b_i_)	95% CI		*p-*Value
			Lower	Upper	
Histology (serous vs. other)	0.211	1.235	0.658	2.317	0.511
Grade (G1 vs. G2, G3)	0.942	2.565	1.290	5.100	0.007
FIGO (I, II vs. III, IV)	1.140	3.126	1.537	6.357	0.002
Patients’ age (≤60 vs. >60 years)	0.312	1.367	0.861	2.169	0.185
Gal-1 stroma (low vs. high)	0.571	1.770	1.044	2.999	0.034
Gal-1 cytoplasm (neg. vs. pos.)	−0.187	0.830	0.423	1.626	0.586
Gal-3 nucleus (neg. vs. pos.)	−0.265	0.767	0.480	1.227	0.269
Gal-7 cytoplasm (neg. vs. pos.)	0.636	1.889	1.160	3.077	0.011

HR = *hazard ratio*; CI = *confidence interval*.

**Table 3:** Gal-3 staining correlated with clinical and pathological data.

Clinical and Pathological Variables	Gal-3 Expression Cytoplasm		*p*	Gal-3 Expression Stroma		*p*	Gal-3 Expression Nucleus		*p*
	neg.	pos.		neg.	pos.		neg.	pos.	
Histology									
Serous	37	69	NS	42	64	0.008	44	62	0.013
Clear cell	3	9		2	10		3	9	
Endometrioid	12	9		13	8		16	5	
Mucinous	3	9		9	3		5	7	
Tumor Stage									
pT1	12	27	NS	21	18	NS	12	27	0.042
pT2+	43	68		44	67		55	56	
Lymph node									
pN0/pNX	39	62	NS	47	54	NS	48	53	NS
pN1	16	34		19	31		20	30	
Distant Metastasis									
pM0/pMX	53	92	NS	64	81	NS	65	80	NS
pM1	2	4		2	4		3	3	
Grading									
G1	9	28	NS	16	21	NS	13	24	NS
G2+	40	62		44	58		51	51	
FIGO									
I/II	13	30	NS	21	22	NS	15	28	NS
III/IV	41	62		43	60		51	52	
Age									
≤60 years	22	57	0.022	33	46	NS	28	51	0.013
>60 years	33	39		33	39		40	32	

TNM staging was accomplished according to actual standards of UICC; pT1 = tumor stage 1; pT2+ = tumor stage 2 or higher; pN0 = lymph node stage 0; pNX = lymph node stage not evaluated; pN1 = lymph node stage 1; pM0 = distant metastasis stage 0; pMX = distant metastasis not evaluated; pM1 = distant metastasis stage 1; G1 = grade 1; G2+ = grade 2 or higher; NS = Not significant (*p* > 0.05).

**Table 4:** Gal-7 staining correlated with clinical and pathological data.

Clinical and Pathological Variables	Gal-7 Expression Cytoplasm			*p*
	neg.	low	high	
Histology				
Serous	10	83	12	0.026
Clear cell	0	10	2	
Endometrioid	7	13	0	
Mucinous	3	8	1	
Tumor Stage				
pT1	4	29	5	NS
pT2+	15	85	10	
Lymph node				
pN0/pNX	15	75	8	NS
pN1	5	39	7	
Distant Metastasis				
pM0/pMX	19	110	14	NS
pM1	1	4	1	
Grading				
G1	6	25	3	NS
G2+	12	80	11	
FIGO				
I/II	8	29	4	NS
III/IV	11	81	11	
Age				
≤60 years	12	59	8	NS
>60 years	8	55	7	

TNM staging was accomplished according to actual standards of UICC; pT1 = tumor stage 1; pT2+ = tumor stage 2 or higher; pN0 = lymph node stage 0; pNX = lymph node stage not evaluated; pN1 = lymph node stage 1; pM0 = distant metastasis stage 0; pMX = distant metastasis not evaluated; pM1 = distant metastasis stage 1; G1 = grade 1; G2+ = grade 2 or higher; NS = Not significant (*p* > 0.05).

**Table 5:** Correlation analysis.

Staining		Gal-1 Cytoplasm	Gal-1 Stroma	Gal-1 Nucleus	Gal-3 Cytoplasm	Gal-3 Stroma	Gal-3 Nucleus	Gal-7 Cytoplasm
Gal-1 cytoplasm								
	cc	1.000	0.382	0.748	0.356	0.263	0.282	0.272
	*p*	.	<0.001	<0.001	<0.001	0.001	<0.001	0.001
	*n*	150	150	150	149	149	149	146
Gal-1 stroma								
	cc	0.382	1.000	0.231	0.123	0.280	−0.006	−0.040
	p	<0.001	.	0.004	0.135	0.001	0.937	0.633
	n	150	150	150	149	149	149	146
Gal-1 nucleus								
	cc	0.748	0.231	1.000	0.302	0.315	0.329	0.249
	*p*	<0.001	0.004	.	<0.001	<0.001	<0.001	0.002
	*n*	150	150	150	149	149	149	146
Gal-3 cytoplasm								
	cc	0.356	0.123	0.302	1.000	0.293	0.839	0.276
	*p*	<0.001	0.135	<0.001	.	<0.001	<0.001	0.001
	*n*	149	149	149	151	151	151	146
Gal-3 stroma								
	cc	0.263	0.280	0.315	0.293	1.000	0.267	0.231
	*p*	0.001	0.001	<0.001	<0.001	.	0.001	0.005
	*n*	149	149	149	151	151	151	146
Gal-3 nucleus								
	cc	0.282	−0.006	0.329	0.839	0.267	1.000	0.335
	*p*	<0.001	0.937	<0.001	<0.001	0.001	.	<0.001
	*n*	149	149	149	151	151	151	146
Gal-7 cytoplasm								
	cc	0.272	−0.040	0.249	0.276	0.231	0.335	1.000
	*p*	0.001	0.633	0.002	0.001	0.005	<0.001	.
	*n*	146	146	146	146	146	146	149

IR scores of Gal-1, -3, and -7 staining in different compartments were correlated with each other using Spearman’s correlation analysis. cc = correlation coefficient, *p* = two-tailed significance, *n* = number of patients.

## Abbreviations

Gal	Galectin
BRCA	Breast cancer gene
CRD	Carbohydrate-recognition domain
UICC	Union for International Cancer Control
FIGO	Fédération Internationale de Gynécologie et d’Obstétrique
IRS	Remmele score
